# Identification of differentially expressed genes and signaling pathways in human conjunctiva and reproductive tract infected with *Chlamydia trachomatis*

**DOI:** 10.1186/s40246-021-00313-8

**Published:** 2021-04-19

**Authors:** Guo-Dong Zhu, Xun-Jie Cao, Ya-Ping Li, Jia-Xin Li, Zi-Jian Leng, Li-Min Xie, Xu-Guang Guo

**Affiliations:** 1 Departments of Geriatrics and Oncology, Guangzhou First People’s Hospital, School of Medicine, South China University of Technology, Guangzhou, 510180 Guangdong China; 2 Department of Clinical Laboratory Medicine, The Third Affiliated Hospital of Guangzhou Medical University, Guangzhou, 510150 China; 3 Department of Clinical Medicine, The Third Clinical School of Guangzhou Medical University, Guangzhou, 511436 China; 4 Department of Clinical Medicine, The Second Clinical School of Guangzhou Medical University, Guangzhou, 511436 China; 5 Key Laboratory for Major Obstetric Diseases of Guangdong Province, The Third Affiliated Hospital of Guangzhou Medical University, Guangzhou, ,510150 China; 6 Key Laboratory of Reproduction and Genetics of Guangdong Higher Education Institutes, The Third Affiliated Hospital of Guangzhou Medical University, Guangzhou, 510150 China

**Keywords:** Conjunctival, Reproductive tract, *Chlamydia trachomatis*, Bioinformatics analysis

## Abstract

**Background:**

Currently, *Chlamydia trachomatis*–specific host defense mechanisms in humans remain poorly defined. To study the characteristics of host cells infected early with *Chlamydia trachomatis*, we used bioinformatics methods to analyze the RNA transcription profiles of the conjunctiva, fallopian tubes, and endometrium in humans infected with *Chlamydia trachomatis*.

**Method:**

The gene expression profiles of GSE20430, GSE20436, GSE26692, and GSE41075 were downloaded from the Gene Expression Synthesis (GEO) database. Then, we obtained the differentially expressed genes (DEGs) through the R 4.0.1 software. STRING was used to construct protein–protein interaction (PPI) networks; then, the Cytoscape 3.7.2 software was used to visualize the PPI and screen hub genes. GraphPad Prism 8.0 software was used to verify the expression of the hub gene. In addition, the gene–miRNA interaction was constructed on the NetworkAnalyst 3.0 platform using the miRTarBase v8.0 database.

**Results:**

A total of 600 and 135 DEGs were screened out in the conjunctival infection group and the reproductive tract infection group, respectively. After constructing a PPI network and verifying the hub genes, CSF2, CD40, and CSF3 in the reproductive tract infection group proved to have considerable statistical significance.

**Conclusion:**

In our research, the key genes in the biological process of reproductive tract infection with *Chlamydia trachomatis* were clarified through bioinformatics analysis. These hub genes may be further used in clinical treatment and clinical diagnosis.

**Supplementary Information:**

The online version contains supplementary material available at 10.1186/s40246-021-00313-8.

## Background

*Chlamydia trachomatis* (*C. trachomatis*, Ct), the causative agent of both the blinding eye disease known as trachoma and of sexually transmitted infections, is an obligate intracellular bacterium whose only natural hosts are humans [1]. The distinct developmental cycle of Ct consists of two phases: an infectious, non-replicative elementary body (EB) and a replicative, non-infectious reticulate body (RB), and the bacteria alternate between these two morphologically distinct forms [2]. The latest data from the World Health Organization show that *Chlamydia trachomatis* is the main cause of bacterial sexually transmitted diseases, with 127 million new cases every year, with almost 60% of these infections occurring in young adults aged 14–24 [3, 4].

Ct is the main pathogen that causes both trachoma and sexually transmitted diseases clinically. Most Ct infections have no obvious symptoms, resulting in potential epidemics and often causing multiple complications [5]. Studies have shown that *Chlamydia trachomatis* infections can cause serious damage to the female reproductive system, including (but not limited to) fallopian tube damage, pelvic inflammatory disease, cervicitis, endometritis, ectopic pregnancy, and ultimately female infertility due to fallopian tube abnormalities [6]. What is more, Ct infection can also cause urethritis, epididymitis, prostatitis, and infertility in men [7]. Moreover, trachoma is caused by repeated infection by *Chlamydia trachomatis*, which leads to the formation of scar tissue on the inner surface of the eyelid, along with the erosion of the surface of the cornea, ultimately leading to blindness [8]. In fact, this disease is still an important cause of blindness worldwide. In addition, *Chlamydia trachomatis* infection increases the risk of chronic fatigue syndrome and reactive arthritis, doubles the risk of ectopic pregnancy, and increases the risk of infection with sexually transmitted diseases such as human papillomavirus and HIV [9].

Gene expression is examined using array technology, which can effectively and simultaneously measure thousands of different mRNAs from a single biological sample [10, 11]. Microarrays can be manufactured by a variety of techniques, including spotting, inkjet synthesis, and photolithographic synthesis [12]. So far, this technology has been applied in many fields [13–15]. Additionally, it can be used to analyze the transcriptome of humans infected with *Chlamydia trachomatis*.

In order to study the characteristics of early host cells infected with *Chlamydia trachomatis*, this study used bioinformatics to analyze the RNA transcription profiles of human conjunctiva, fallopian tube, and endometrial tissue infected by *Chlamydia trachomatis*.

## Materials and methods

### Data source

The datasets analyzed in this study were all from the GEO database (https://www.ncbi.nlm.nih.gov/geo/) after searching for keywords related to *Chlamydia trachomatis*. Finally, 79 series about Ct were obtained. We selected four separate gene expression profiles (GSE20430, GSE20436, GSE26692, and GSE41075) for our study. Information about 119 human samples of *Chlamydia trachomatis* infection was retrieved from these profiles. Among them, GSE20430 was based on the GPL201platform, GSE20436 was based on GPL570, GSE26692 was based on GPL4133, and GSE41075 was based on GPL571.

### Data processing and identification of DEGs

Using the R 4.0.1 software, the data of GSE26692 was batch calibrated and standardized via the *marray* package. The data of GSE20430, GSE20436, and GSE41075 were batch calibrated and standardized by using the *affy* package. Then, the *limma* package was applied to identify the differentially expressed genes (DEGs). In addition, the *ggpubr* and *ggthemes* packages were used to draw volcano maps, and the *pheatmap* software package was used to draw heatmaps to visualize the DEGs. When analyzing the data of GSE26692 and GSE41075, *P* < 0.2 and |FC| ≥ 1 were used as critical values to compare the gene expression profiles of infected and uninfected samples. The data of GSE20430 and GSE20436 were analyzed with *P* < 0.05 and |FC| ≥ 1 as critical values.

### GO and KEGG pathway analysis of DEGs

The Gene Ontology (GO) database was used to compile the functional analysis of DEGs in terms of molecular function (MF), biological process (BP), and cellular component (CC). The Kyoto Encyclopedia of Genes and Genomes (KEGG) was used to investigate the signaling pathways of DEGs. The GO and KEGG pathway analyses of the DEGs were performed using the cluster Profiler package in the R software (*P* value cutoff = 0.05).

### PPI network construction and hub gene identification

We used the Search Tool for the Retrieval of Interacting Genes (STRING), a database analysis platform, to analyze our DEGs data and obtain a protein–protein interaction (PPI) map. With respect to the PPI, pairs with a combined score > 0.4 were selected to visualize the PPI network using Cytoscape 3.7.2 software. The degree of node connection is positively correlated with the stability of the whole network. In addition, we selected the top 10 genes in the central index as the core candidate genes.

### Verification of intersection hub genes and construction of intersection gene–miRNA interaction

The DEGs between the normal (non-infected) samples and conjunctiva samples infected by *Chlamydia trachomatis* were identified by data from two transcription profiles (GSE20430 and GSE20436). The DEGs between the normal samples and reproductive tract samples infected by *Chlamydia trachomatis* were identified by data from two other transcription profiles (GSE26692 and GSE41075). The tool used for analysis was *OmicShare Tools* (https://www.omicshare.com/tools/Home/Soft/venn). After obtaining two sets of hub genes, GSE87110 was used to verify the hub genes of the reproductive tract infection. However, the hub genes of the conjunctival infection could not be verified, since there was no suitable dataset. The above analysis was done by using the GraphPad Prism 8.0 software. In addition, the gene–miRNA interaction was constructed on the NetworkAnalyst 3.0 platform using the miRTarBase v8.0 database (https://www.networkanalyst.ca/NetworkAnalyst/home.xhtml).

## Results

### Identification of DEGs

**Fig. 1:**
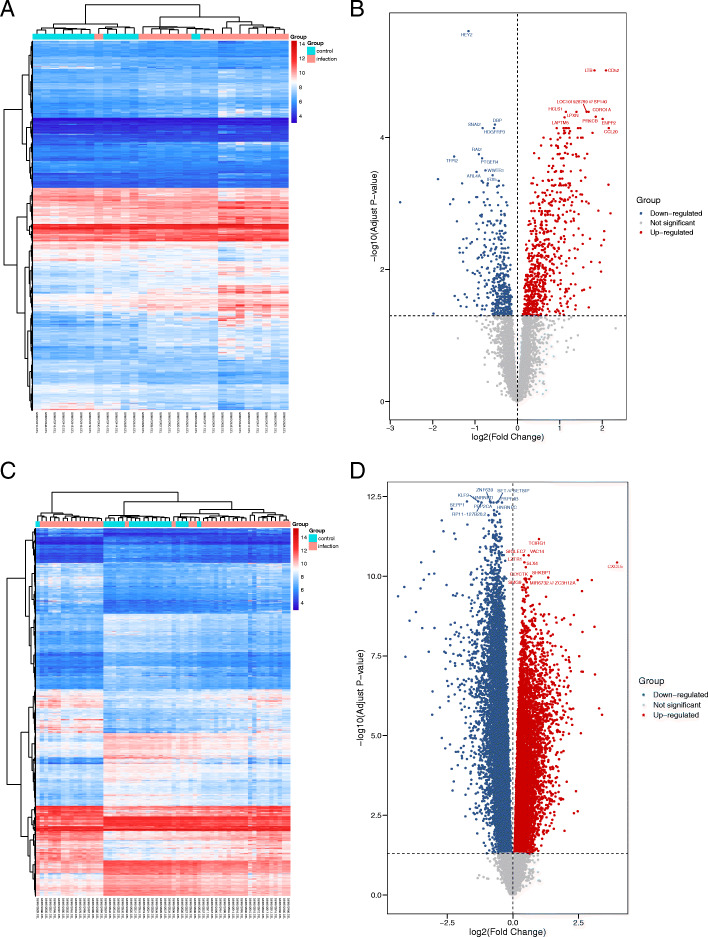
Volcano map and heat map of differentially expressed genes (DEGs) in GSE20430 and GSE20436. Volcano map in GSE20430 (**a**) and heat map in GSE20430 (**b**). Volcano map in GSE20436 (**c**) and heat map in GSE20436 (**d**)

**Fig. 2:**
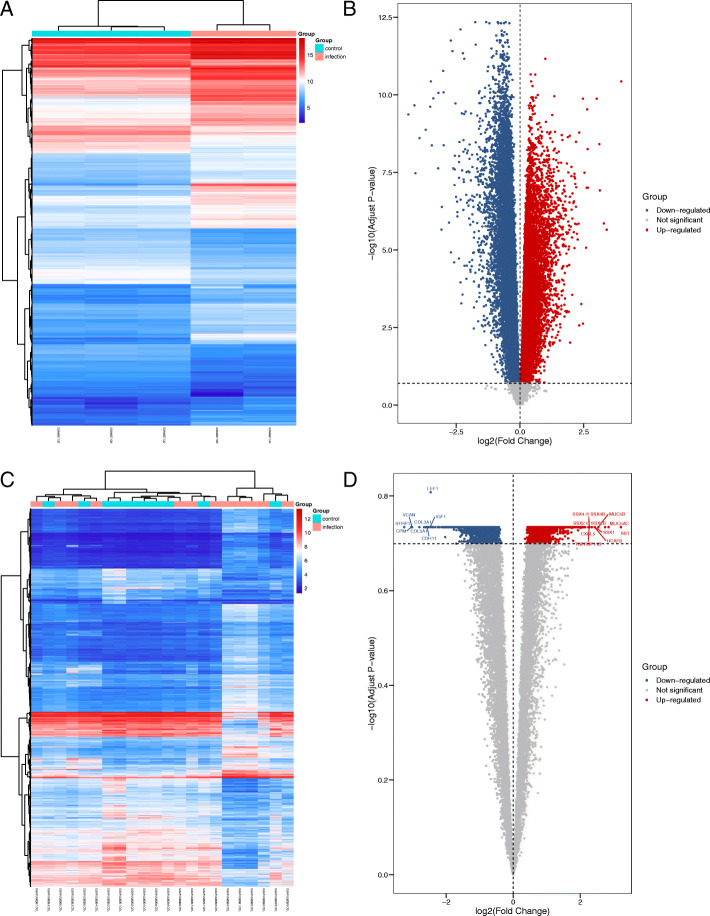
Volcano map and heat map of differentially expressed genes (DEGs) in GSE26692 and GSE41075. Volcano map in GSE26692 (**a**) and heat map in GSE26692 (**b**). Volcano map in GSE41075 (**c**) and heat map in GSE41075 (**d**)

In the GSE20430 data set, 29 samples were included, including 12 controls. The GSE20436 data set included 60 samples, including 20 controls. Five and 22 samples were included in the GSE26692 and GSE41075 datasets, respectively, with 3 and 10 controls, respectively. The two conjunctival infection groups showed 1104 and 4159 DEGs, respectively. The fallopian tube infection group and the endometrial infection group obtained 12441 DEGs and 6744 DEGs, respectively. Volcano maps and heatmaps were used to visualize the differences in gene expression. The gene expression profiles of infected conjunctiva were GSE20430 (Fig. 1a and 1b) and GSE20436 (Fig. 1c and 1d); the gene expression profile of infected fallopian tube epithelial cells was GSE26692 (Fig. 2a). The gene expression profile of infected endometrial epithelial cells was GSE41075 (Fig. 2c and 2d).

### GO enrichment analysis of DEGs

**Fig. 3:**
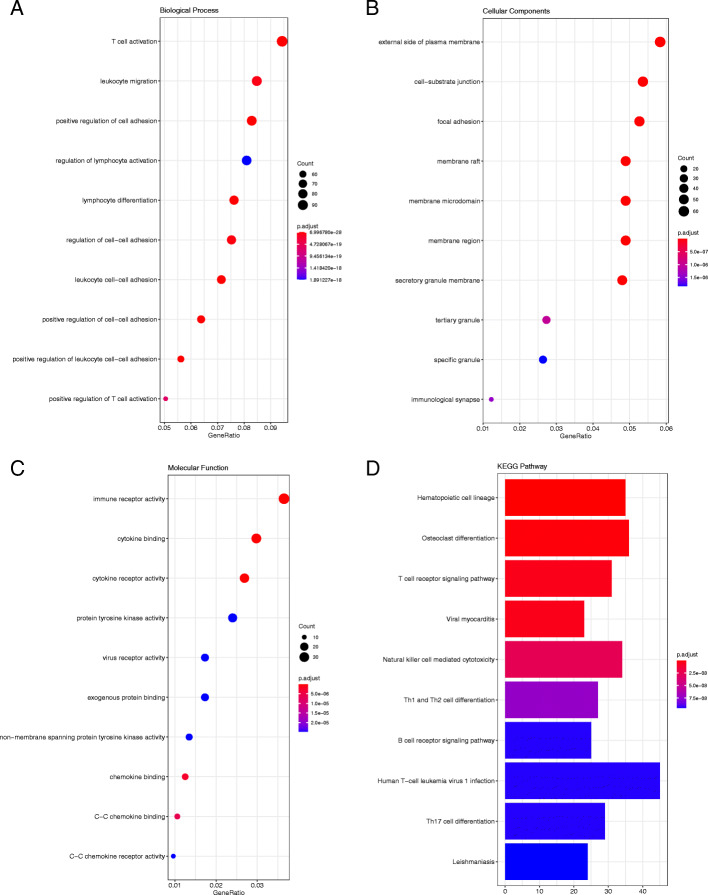
The GO enrichment analysis and KEGG pathway analysis of DEGs for GSE20430. Biological Process (**a**), Cellular Component (**b**), Molecular Function (**c**) and KEGG pathway analysis (**d**)

The GO functional enrichment analysis was performed for GSE20430, GSE20436, GSE26692, and GSE41075 gene expression profiles. With respect to the GSE20430 dataset (Fig. 3), the results of CC showed that the DEGs were mainly enriched at the external side of the plasma membrane, cell–substrate junction, and focal adhesion. As for BP, the results indicated that the DEGs were principally enriched in T cell activation, leukocyte migration, and positive regulation of cell adhesion. The results of the MF analysis showed that the DEGs were significantly rich in immune receptor activity, cytokine binding, and cytokine receptor activity.

**Fig. 4:**
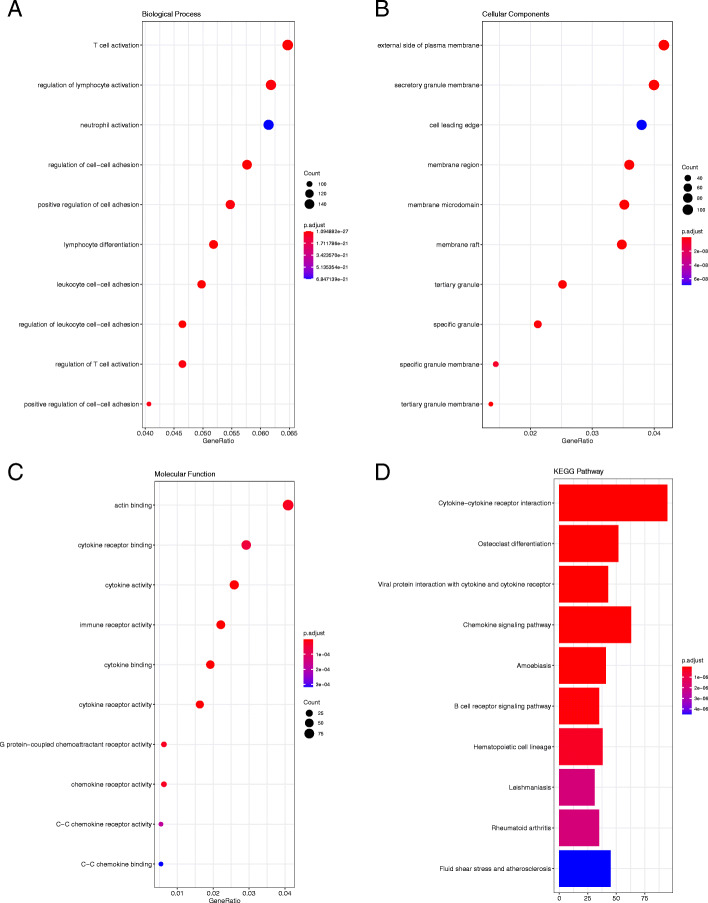
The GO enrichment analysis and KEGG pathway analysis of DEGs for GSE20436. Biological Process (**a**), Cellular Component (**b**), Molecular Function (**c**) and KEGG pathway analysis (**d**)

In the context of the GSE20436 dataset (Fig. 4), the DEGs in BP were enriched in T cell activation, regulation of lymphocyte activation, and regulation of cell−cell adhesion; in MF, they were enriched in actin binding, cytokine receptor, and binding cytokine activity; and in CC, they were enriched in the external side of plasma membrane, the secretory granule membrane, and membrane region.

**Fig. 5:**
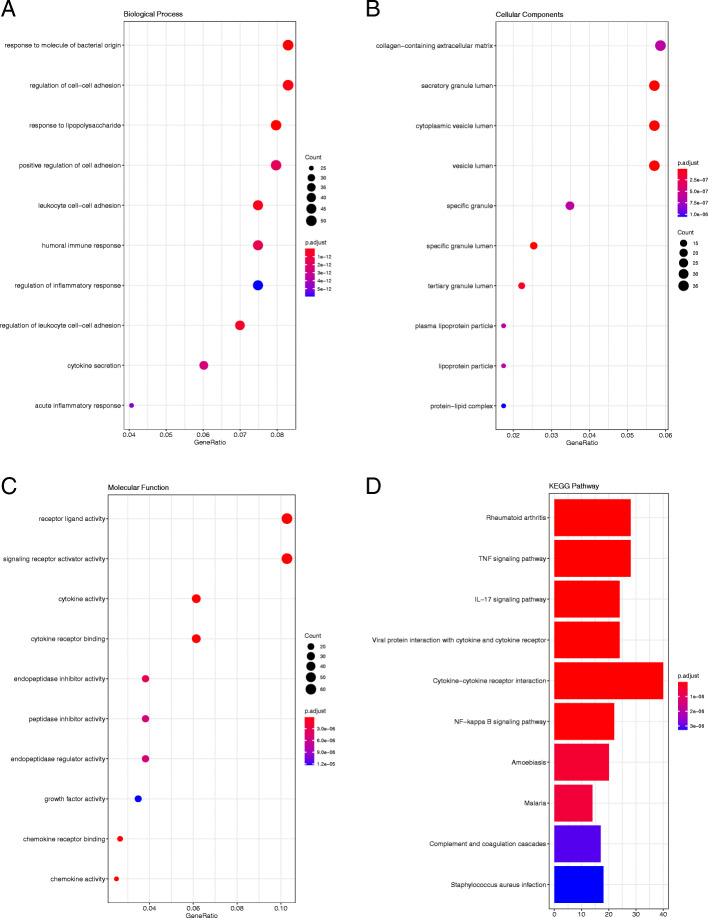
The GO enrichment analysis and KEGG pathway analysis of DEGs for GSE26692. Biological Process (**a**), Cellular Component (**b**), Molecular Function (**c**) and KEGG pathway analysis (**d**)

With respect to the GSE26692 dataset (Fig. 5), it was observed that the DEGs in BP were enriched in response to molecules of bacterial origin, regulation of cell−cell adhesion, and response to lipopolysaccharides; in MF, they were enriched in receptor ligand activity, signaling receptor activator activity, and cytokine activity; and in CC, they were enriched in collagen-containing extracellular matrix, secretory granule lumen, and cytoplasmic vesicle lumen.

**Fig. 6:**
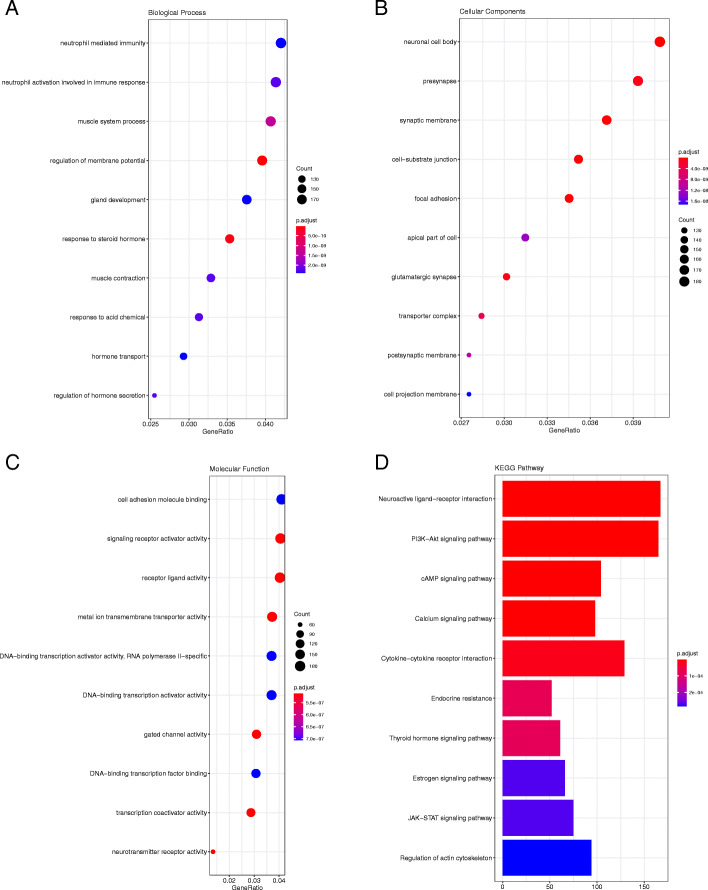
The GO enrichment analysis and KEGG pathway analysis of DEGs for GSE41075. Biological Process (**a**), Cellular Component (**b**), Molecular Function (**c**) and KEGG pathway analysis (**d**)

In relation to the GSE41075 dataset (Fig. 6), it was observed that the DEGs in BP were enriched in neutrophil activation involved in immune response, neutrophil-mediated immunity, and regulation of membrane potential; in MF, they were enriched in signaling receptor activator activity, receptor ligand activity, and cell adhesion molecule binding; in CC, they were enriched in neuronal cell body, presynapse, and synaptic membrane.

### KEGG pathway enrichment analysis

In order to better identify the biological functions of the DEGs, a KEGG pathway analysis was conducted. *P* < 0.05 was considered statistically significant. The results of the analysis are shown in Figs. 3, 4, 5 and 6. According to the *P* value, ten significant enrichment pathways of two conjunctival infection groups, one fallopian tube infection group, and one endometrial infection group were obtained. The significant enrichment pathways found in case of the conjunctival infection groups are shown in Figs. 3 and 4, respectively. The significant enrichment pathways found in fallopian tube infections and endometrial infections are shown in Figs. 5 and 6, respectively.

### Construction of PPI network and hub genes identification

**Fig. 7:**
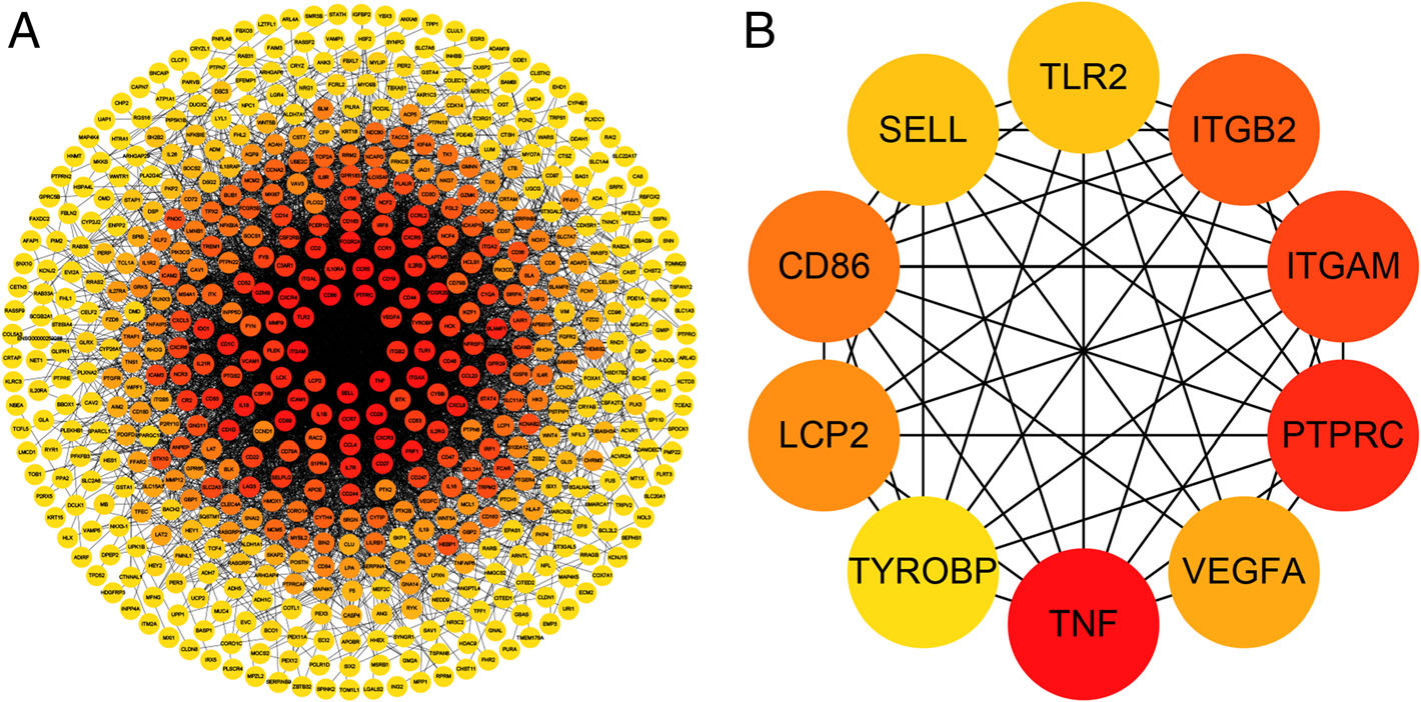
The PPI network of overlapping DEGs in GSE20430 and GSE20436 (**a**) and the important module of PPI network (**b**)

The intersection genes of GSE20430 and GSE20436 with respect to conjunctiva (Figure S1) were obtained. In order to study the relationship between different gene expression proteins in conjunctival intersection genes, we uploaded 600 DEGs to STRING to establish a PPI network (Fig. 7a). The PPI network involved a total of 599 nodes and 5595 edges. The hub genes are shown in Fig. 7b.

**Fig. 8:**
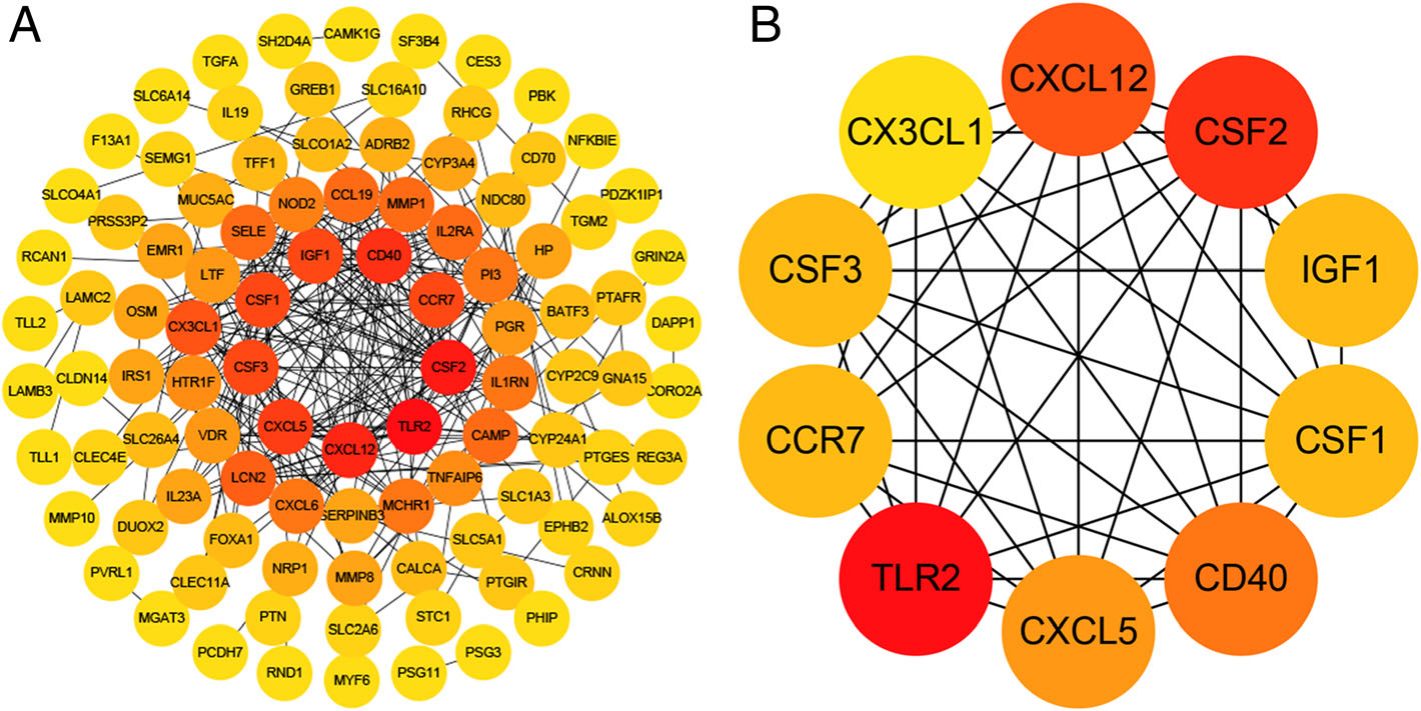
The PPI network of overlapping DEGs in GSE26692 and GSE41075 (**a**) and the important module of PPI network (**b**)

Then, the intersection genes of GSE26692 and GSE41075 in reproductive tract infection were obtained (Figure S2). In order to study the relationship between different gene expression proteins in this group of intersection genes, we uploaded 135 DEGs to STRING to establish a PPI network (Fig. 8a). The PPI network involved a total of 131 nodes and 262 edges. The hub genes are shown in Fig. 8b.

### Verification of intersection hub genes and construction of intersection gene–miRNA interaction

**Fig. 9:**
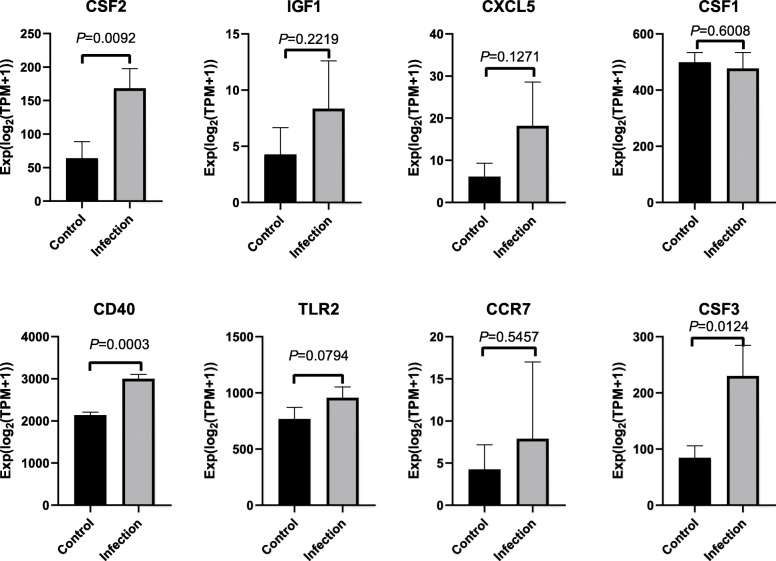
Verification of hub genes. P-value > 0.05 is considered to be statistically significant

**Fig. 10:**
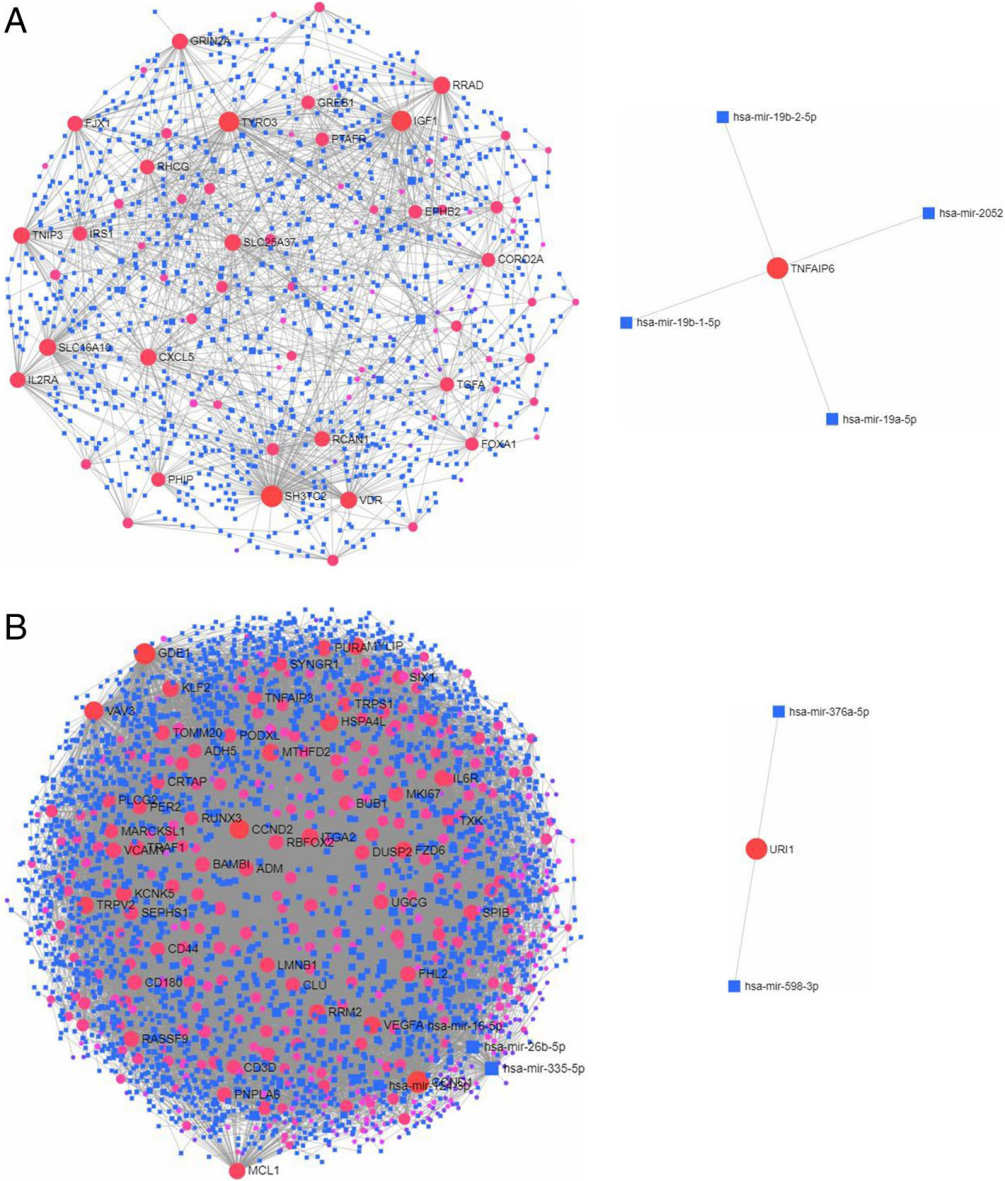
The miRNA-target gene network of overlapping DEGs. Gene–miRNA interaction for the intersection genes of reproductive tract infection (**a**) and gene-miRNA interaction for the intersection genes of conjunctival infection (**b**)

In order to make the research more rigorous, we used GSE87110 to verify the hub genes of reproductive tract infections. With *P* < 0.05 as the threshold, we found that the analysis results of the hub genes (CSF2, CD40, and CSF3) were statistically significant (Fig. 9). The gene–miRNA interaction of the two sets of intersection genes is shown in Fig. 10. There were two subnetworks for gene–miRNA interaction for the intersection genes of reproductive tract infection and conjunctival infection. The top 5 miRNA ranked by degree of significance in case of reproductive tract infection and conjunctival infection are shown in Table 1 and Table 2, respectively.

**Table 1:**
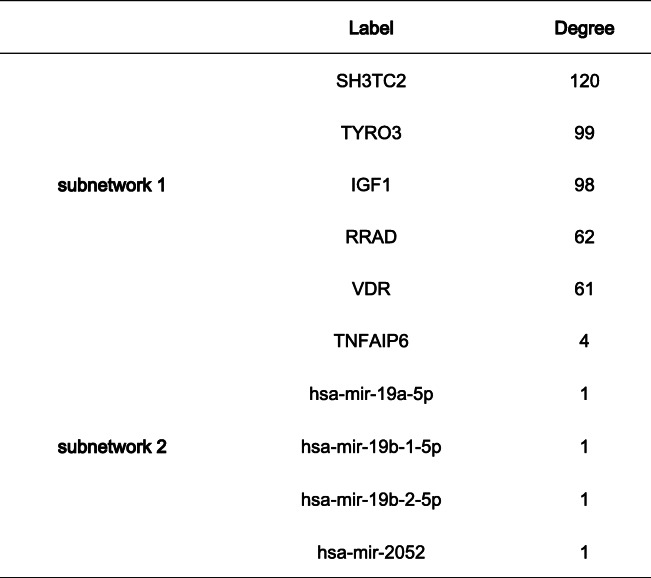
The top 5 miRNA ranked by degree in reproductive tract infection

**Table 2:**
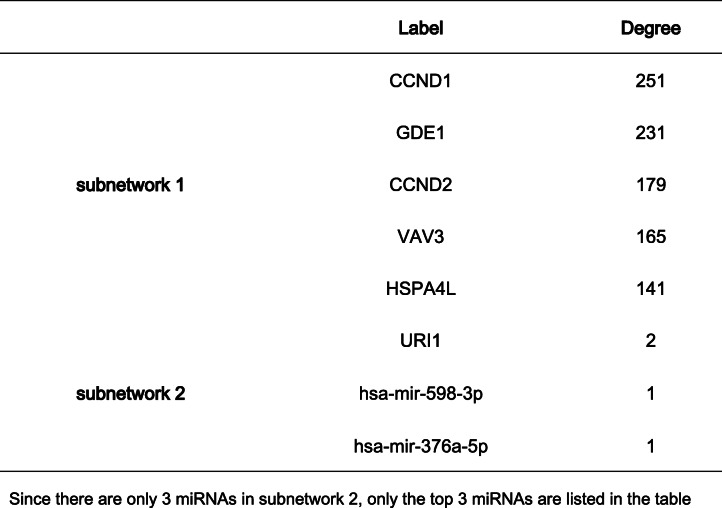
The top 5 miRNA ranked by degree in conjunctival infection

## Discussion

Bioinformatics concepts were applied in this research to analyze the RNA transcription profiles of human conjunctiva, fallopian tube, and endometrial epithelial cells infected by Ct to study the characteristics of early infected host cells.

According to the GO enrichment analyses, regulation of T cell activation showed high enrichment scores in the BP among the conjunctival infection group, which corresponded to the previous findings that CD4 T cells and IFN-γ play a primary role in immunity against Ct infection [16]. Interestingly, in the endometrium group, the BP was observed to be significantly associated with neutrophil activation involved in immune response. In 2018, Karthika Rajeeve et al. reported that *Chlamydia trachomatis* paralyzes neutrophils to evade the host’s innate immune response, implying that neutrophil activation is essential to the anti-infectious immunity [17]. Besides, the MF of the two groups of conjunctiva and fallopian tubes were enriched in cytokine receptor activity, including interferon-class cytokine receptor activity and IL receptor interleukin receptor activity. Numerous studies have confirmed the importance of cytokines such as IFN-γ in host resistance to Ct infection [18]. Among them, multiple interferon-stimulated genes (ISG)–mediated cell-autonomous host defenses have been shown to protect mice against experimental Ct infection [19]. In the fallopian tube infection group and endometrium infection group, the MF were highly associated with receptor ligand activity, including the vitamin D receptor activator activity, suggesting its involvement in defense against Ct infections [20].

In the current study, KEGG was used to identify certain cell signaling pathways that are closely related to Ct infection. In this study, the common enrichment pathways of the two conjunctival data sets are the hematopoietic cell lineage pathway and the osteoclast differentiation pathway. Studies have shown that the blood system has a high response ability to generate inflammation signals caused by infection or injury and hematopoietic stem cells are responsible for the final production of blood cells and play an important role in immunity and tissue repair [21]. The hematopoietic cell lineage pathway plays an important role in early infections. Therefore, hematopoietic cell lineage pathway plays a “first responder” role in host defense when *Chlamydia trachomatis* infects conjunctival epithelial cells. In addition, Ct infects conjunctival epithelial cells to promote osteoclast differentiation, and its pathway is achieved through the PI3K-Akt signaling pathway and the cAMP signaling pathway [22]. These two signaling pathways were also enriched in the fallopian tube infection in this study. Many studies have determined that the PI3K-Akt signaling pathway plays an important role in the differentiation and function of osteoclasts [22]. Osteoclasts play an absorption role in local inflammatory lesions. Since cAMP has a regulatory effect on the transcription level of *Chlamydia trachomatis*, it has an inhibitory effect on the development cycle of *Chlamydia trachomatis* [23, 24]. Therefore, it can be inferred that the osteoclast differentiation pathway has the effect of resisting infection aggravation and immune clearance in Ct infection of the conjunctiva. Additionally, the results of this study show that the interaction pathways of cytokines and cytokine receptors are enriched in infections of the conjunctiva, fallopian tube, and endometrium. Studies have shown that the interaction between cytokines and receptors may be crucial for determining the role of inflammation in the development of diseases, because after the cells are infected, the host cells induce a large number of cytokines, chemokines, and reactive oxygen species. This in turn activates natural immunity and regulatory immune response and, through the interaction of cytokines and cytokine receptors, play a role in the clearance process [25]. Therefore, in Ct infection of conjunctival and genital epithelial cells, the interaction of cytokines and cytokine receptors may play a role in immune response and immune clearance.

Determining the relationship between proteins is an important step in understanding protein functions and identifying related biological pathways [26]. A growing body of evidence shows that protein–protein interactions are critical in many biological processes in living cells [27]. Therefore, the hub gene selected by PPI may also play a crucial role in the biological process of Ct infection. We used GSE87110 to verify the hub genes in case of reproductive tract infections. Then, we found that the CSF2, CD40, and CSF3 genes were statistically significant. Studies have shown that epithelial cells infected by *Chlamydia trachomatis* can release CSF2, which can mediate the influx and activation of inflammatory cells at the infection site [28, 29]. In addition, the secretion of CSF2 can promote the maturation and activation of neutrophils [30]. Therefore, CSF2 plays an important role in the inflammatory response. In Schlievert et al.’s study of *Staphylococcus aureus* infection, it was found that superantigens can destroy the mucosal barrier by binding to CD40 and then express chemokines to promote infection [31]. Therefore, we can infer that the upregulation of CD40 means that such a mechanism may also exist in Ct infection. Like CSF2, CSF3 is also involved in the host response to microbial infections [32]. CSF3 can increase the chemotactic activity of neutrophils, thus causing inflammation [33].

In this study, we identified the key genes for Ct infection in the reproductive tract and conjunctiva. However, we could only verify the key genes of reproductive tract infection through external dataset. More experiments need to be implemented to verify these key genes in the conjunctival cells as well.

## Conclusion

In our research, the key genes in the biological process of reproductive tract infection with *Chlamydia trachomatis* were clarified through bioinformatics analysis. These hub genes mainly affect the verification process of *Chlamydia trachomatis* infection and may be further used in clinical treatment and clinical diagnosis.

## Supplementary Information

**Additional file 1: Figure S1**. The intersection results of GSE20430 and GSE20436

**Additional file 2: Figure S2**. The intersection results of GSE26692 and GSE41075

## Data Availability

The datasets used and/or analyzed during the current study are available from the corresponding author on reasonable request.

## Abbreviations

GEO: Gene Expression Synthesis
DEGs: Differentially expressed genes
PPI: Protein–protein interaction
*C. trachomatis*, Ct: *Chlamydia trachomatis*
EB: Elementary body
RB: Reticulate body
GO: Gene ontology
MF: Molecular function
BP: Biological process
CC: Cellular component
KEGG: Kyoto Encyclopedia of Genes and Genomes
STRING: Search Tool for the Retrieval of Interacting Genes
ISG: Interferon-stimulated genes
