# Supplementary figures and images for: Identification of differentially expressed genes and signaling pathways in human conjunctiva and reproductive tract infected with Chlamydia trachomatis
Source: Hum Genomics. 2021 Apr 19;15:22. doi: 10.1186/s40246-021-00313-8 (PMC8056519; doi:10.1186/s40246-021-00313-8)

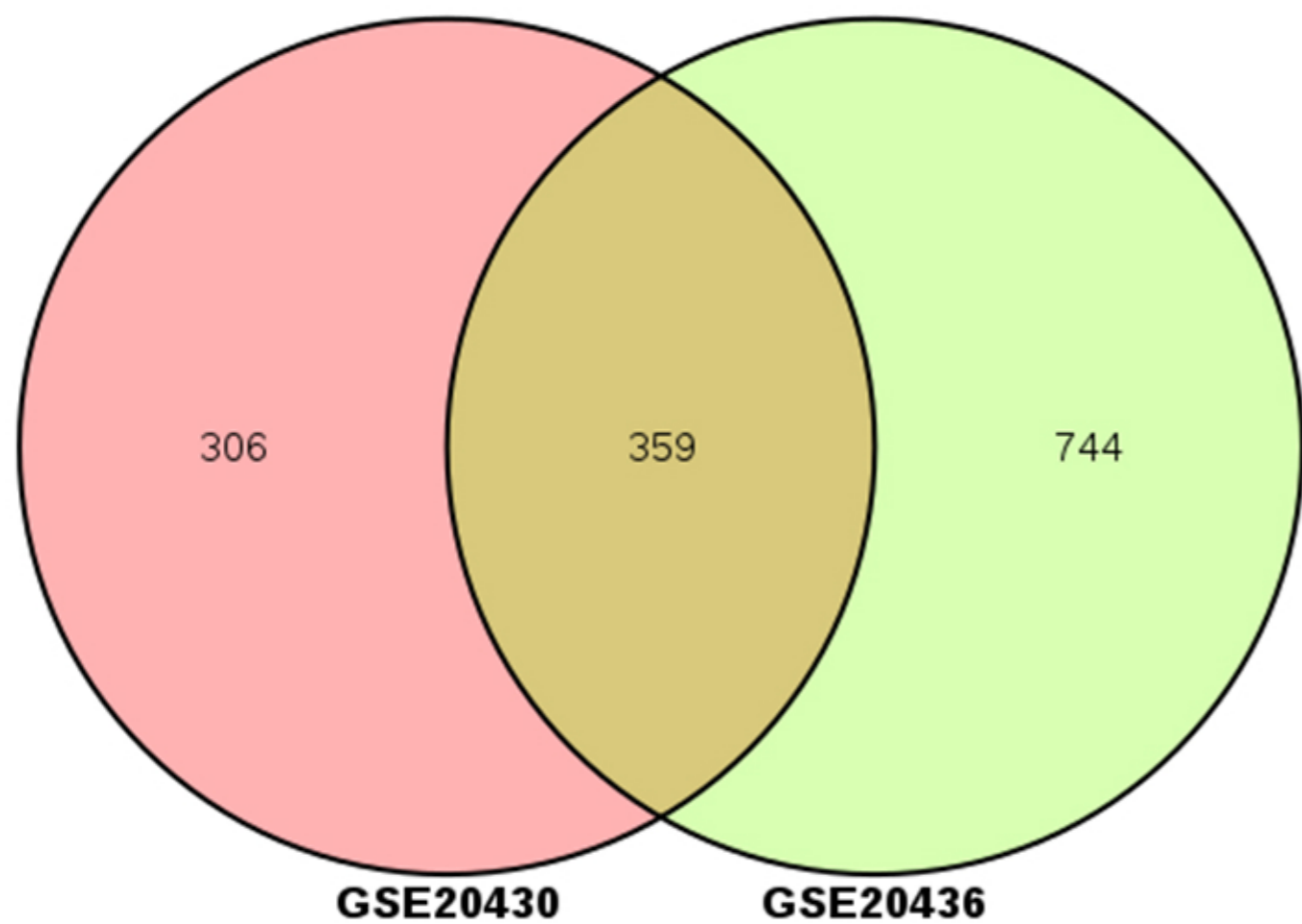

up-regulation

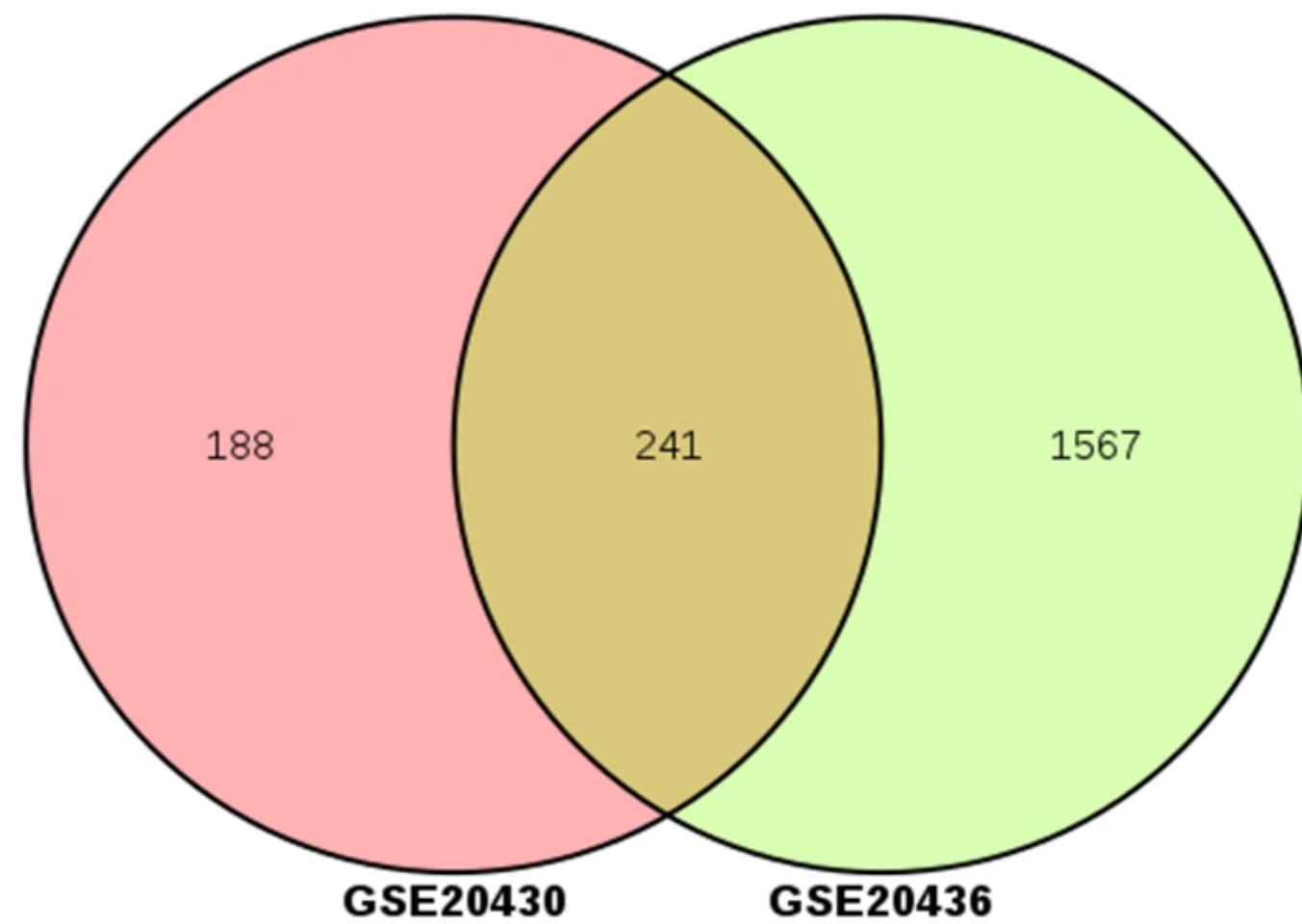

down-regulation

Supplement: Supplementary file 1 — Additional file 1: Figure S1. The intersection results of GSE20430 and GSE20436 [file 40246_2021_313_MOESM1_ESM.pdf]

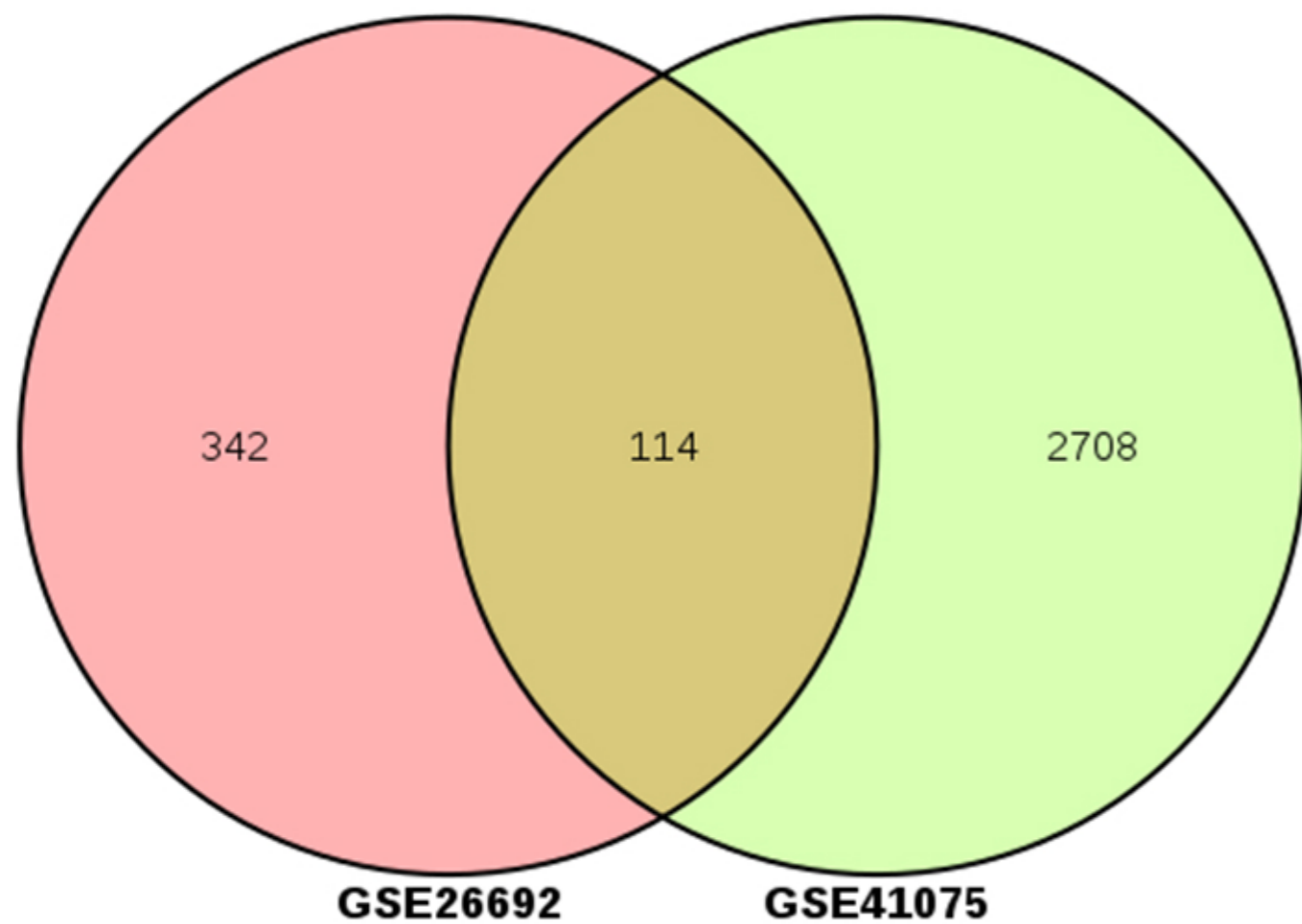

up-regulation

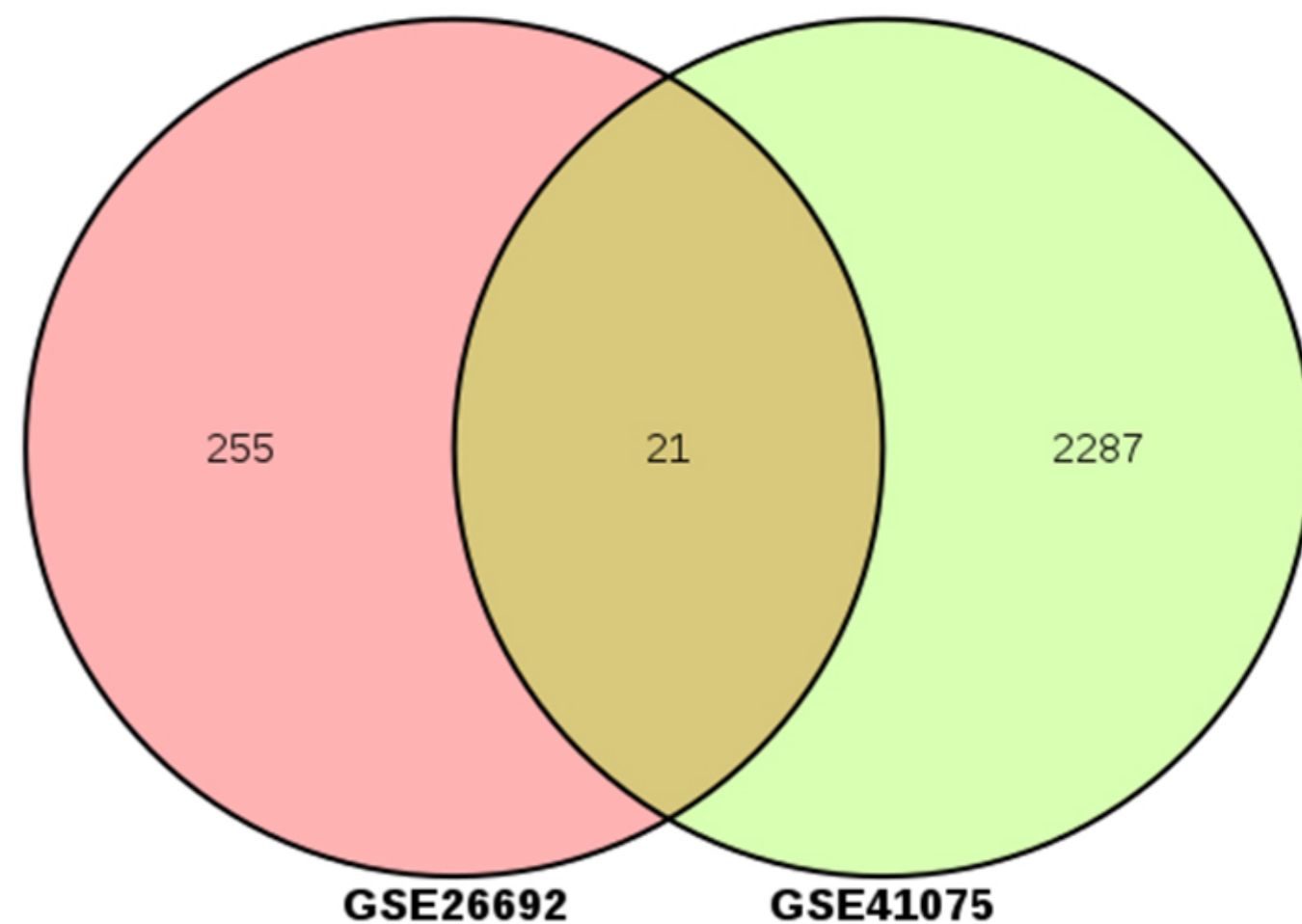

down-regulation

Supplement: Supplementary file 2 — Additional file 2: Figure S2. The intersection results of GSE26692 and GSE41075 [file 40246_2021_313_MOESM2_ESM.pdf]
